# Efficient Green Light‐Induced Photoclick Reactions Enabled by Triplet–Triplet Annihilation Photon Upconversion

**DOI:** 10.1002/anie.8317220

**Published:** 2026-09-29

**Authors:** Qingxin Luan, Mei Sang, Yuxin He, Hao Yang, Anna M. Doze, Lili Hou, Youxin Fu, Ben L. Feringa

**Affiliations:** ^1^ College of Science Nanjing Forestry University Nanjing China; ^2^ State Key Laboratory of Precision Measurement Technology and Instruments School of Precision Instruments and Optoelectronics Engineering Tianjin University Tianjin China; ^3^ Centre For Systems Chemistry Stratingh Institute for Chemistry Faculty For Science and Engineering University of Groningen Groningen The Netherlands

**Keywords:** 9,10‐phenanthrenequinone‐electron‐rich alkenes, low incoherent light intensity, photochemistry, photoclick, triplet–triplet annihilation photon upconversion

## Abstract

Photoclick reactions, combining the advantages of photochemistry and classical click chemistry, are highly promising for surface functionalization, polymer conjugation, and biolabeling. Conventional photoclick reactions rely on the use of high‐energy ultraviolet (UV) light, which suffers from limited penetration depth and cell toxicity. Herein, we proposed a general strategy of efficient green light‐induced photoclick reactions by introducing a triplet–triplet annihilation photon upconversion (TTA‐UC) system. The TTA‐UC system comprises (octaethylporphyrinato) palladium (II) (**PdOEP**) as the sensitizer and 9,10‐diphenylanthracene (**DPA**) as the annihilator, and can effectively convert green light into blue light under incoherent and subsolar light intensity, which can further trigger a series of photoclick reactions based on 9,10‐phenanthrenequinone and electron‐rich alkenes (**PQ‐ERA**s). The pseudo‐first‐order kinetics (*k_obs_*) can reach up to 0.015 s^−1^ under 550 nm LED irradiation (50 mW cm^−2^). This study provides a new approach for realizing photoclick reactions under low‐power, long‐wavelength, and incoherent light excitation, holding great potential for applications in biological and materials systems.

## Graphical abstract

A general strategy for 9,10‐phenanthrenequinone‐electron‐rich alkene (**PQ‐ERA**) photoclick reactions via triplet–triplet annihilation upconversion (**TTA‐UC**) under low‐intensity green light is reported. A **TTA‐UC** system of **PdOEP** (sensitizer) and **DPA** (annihilator) mixed with reactants generates UV/blue photons overlapping the absorption of **PQ** derivatives, enabling fast and efficient reactions, completed within 5 min; rate constant up to 0.014 s^−1,^ under weak 550 nm LED irradiation.

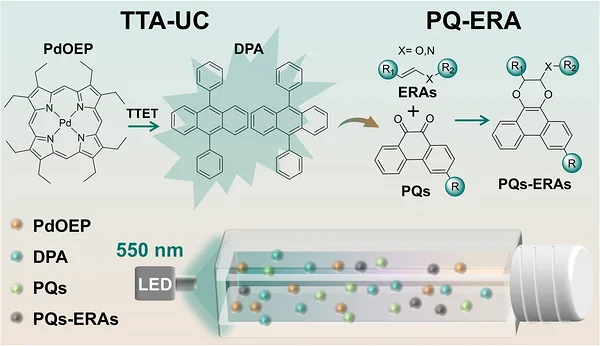

## 1 Introduction

Photoclick chemistry [1, 2, 3], combining click chemistry [4, 5, 6, 7], and photochemical processes [8], enables the rapid and accurate synthesis of various organic structures under mild conditions upon suitable light irradiation, exhibits high precision, and spatiotemporal controllability [1, 2, 9]. Since it was first reported by Lin and coworkers in 2008 [10], the field has undergone rapid development in the past decades, and photoclick approaches [1, 2, 3, 4] have been widely applied in surface functionalization [11, 12, 13, 14], polymer conjugation [15, 16, 17], photocross‐linking [18], and biolabeling in the native cellular environment [19, 20, 21]. Compared to thermally driven reactions, photoclick reactions typically are non‐invasive, exhibiting high yields, short reaction times, excellent functional group orthogonality, and selectivity [22, 23, 24], without the need for acids, bases, or transition‐metal catalysts [1]. Moreover, the use of light provides unparalleled spatial and temporal control, making photoclick chemistry highly promising for applications in chemical biology and materials science, particularly for processes at the single‐cell and nanoscale levels [25, 26, 27].

However, most photoclick reactions require the use of high‐energy photons, such as ultraviolet (UV), violet, or deep blue light to trigger the reactions. For instance, the typical 9, 10‐phenanthrenequinone‐electron‐rich alkenes (**PQ‐ERA**s) photocycloaddition is activated under light irradiation with a wavelength shorter than 450 nm (Scheme 1a, i) [3, 28, 29, 30, 31, 32, 33, 34], which is inherently limited by the absorption band of **PQ** derivatives, typically located in the UV and deep‐blue regions. To avoid the need for high‐energy photons, we recently developed several strategies for visible and near‐infrared (NIR) light‐induced photoclick reactions [3, 29, 30, 31, 32, 33, 35]. The most common approach is red‐shifting the absorption band of reactants into the visible region through π‐conjugation engineering [33, 36, 37]. However, this strategy generally requires complex synthetic efforts and often suffers from low molar extinction coefficients or loss of photoreactivity [28, 38, 39, 40]. Another strategy is indirect sensitization through a triplet–triplet energy transfer (TTET) approach (Scheme 1a, ii) [32]. However, achieving high efficiency in such systems necessitates precise triplet energy alignment between the sensitizer (T_1_) and the photoactive precursors, while the simultaneous modulation of both the triplet energy levels and absorption profiles in organic sensitizers remains a persistent synthetic and materials challenge. Alternatively, upconversion nanoparticles (UCNPs) have recently been employed to enable photoclick reactions driven under NIR light (Scheme 1a, iii) [29]. However, the upconversion quantum yield (UCQY) of UCNPs in the UV and blue region remains low (< 1%) [41, 42, 43], and the operation demands for the use of high‐power coherent laser sources (>8 W cm^−2^). These combined requirements thus restrict their practical application in biological and materials systems [44, 45, 46]. Triplet–triplet annihilation photon upconversion (TTA‐UC) enables the conversion of low‐energy photons to high‐energy ones with high efficiency over 10% under incoherent subsolar light irradiation (<0.1 W cm^−2^) [47, 48], which has been widely applied to diverse fields such as photoredox catalysis, photoactivation, and 3D printing [49, 50]. Compared with reported strategies (Table S1), the TTA‐UC approach is particularly attractive for driving photoclick reactions, because the high‐energy photons can efficiently be generated from singlet excited states through the triplet–triplet annihilation (TTA) process under mild, low‐power light excitation. Importantly, this mechanism decouples light harvesting from the molecular structure of the reactants and without the need for precise triplet energy alignment with **PQ** substrates.

**SCHEME 1:**
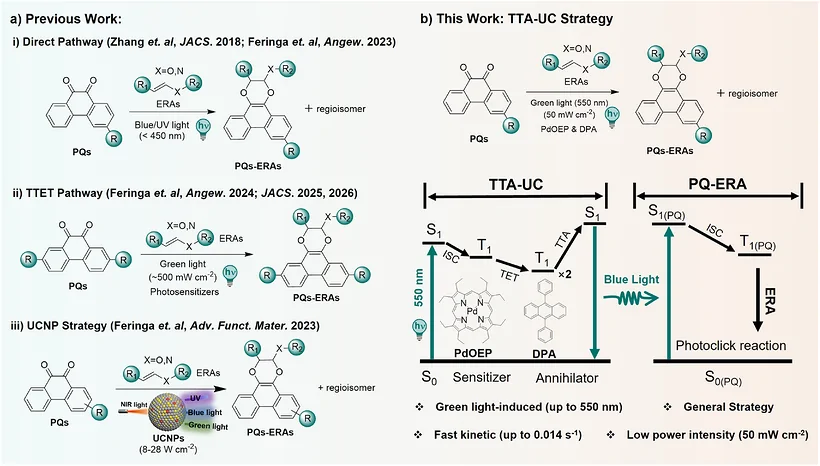
Schematic illustration of photocycloaddition of 9,10‐phenanthrenequinone (**PQ**) with electron‐rich alkenes (**ERA**s). (a) Previous Work: (i) Direct excitation pathway; (ii) TTET pathway; (iii) UCNP strategy, (b) TTA‐UC strategy.

Herein, we report the first demonstration that TTA‐UC can drive the **PQ‐ERA** photoclick reaction by using a low‐power (50 mW cm^−2^), incoherent 550 nm LED light source (Scheme 1b). A conventional TTA‐UC system, consisting of (octaethylporphyrinato) palladium (II) (**PdOEP**) as the sensitizer and 9,10‐diphenylanthracene (**DPA**) as the annihilator [51], was employed, exhibiting an UCQY of up to 17.2%. Low‐energy green photons are absorbed by **PdOEP**, followed by intersystem crossing (ISC) and TTET to **DPA**. Subsequent TTA between two **DPA** molecules generates high‐energy blue emission, which is directly reabsorbed by **PQ** derivatives, thereby efficiently activating the **PQ‐ERA** photoclick reaction. Notably, this TTA‐UC strategy is broadly applicable to successfully activate different alkene substrates and various **PQ** derivatives functionalized with either electron‐donating or electron‐withdrawing substituents. All reactions reach completion within minutes, highlighting the efficiency and versatility of this TTA‐UC‐enabled photoclick approach. Importantly, an alternative green‐to‐blue TTA‐UC pair, based on iodinated boron dipyrromethene (**BODIPY**) sensitizer and perylene annihilator [52], can also drive the **PQ‐ERA** process, demonstrating TTA‐UC approach provides a general and powerful toolbox for expanding the scope of photoclick chemistry. Furthermore, we demonstrate that this TTA‐UC photoclick system can be transferred into water via simple **PEG** encapsulation and remains active in buffers of different pH values and in hydrogels, highlighting its potential for future biological applications.

## 2 Results and Discussion

Commercially available **PdOEP** and **DPA** were used as received for the TTA‐UC system, and the absorption and emission spectra of the individual components and the mixture are shown in Figure 1a. **PdOEP** exhibits a Soret band at 394 nm and two Q bands at 513 and 547 nm, with the latter enabling direct green‐light absorption. Its phosphorescence appears in the red region (590–730 nm), which is positioned outside the absorption range of **PQ** derivatives. Notably, the T_1_ energy level of **PdOEP** (1.87 eV) [53] lies below that of **PQ** derivatives (typically >2.13 eV) [32], precluding direct triplet energy transfer to **PQ** and thus preventing direct photoclick reaction via sensitization. In contrast, **DPA** displays strong absorption between 300 and 420 nm but lacks absorption in the green region. Its fluorescence, however, covers the violet‐blue region (up to 385 nm), which overlaps effectively with the absorption profile of **PQ** molecules. To achieve green‐to‐blue upconversion, a mixture of **PdOEP** (30 µM) and **DPA** (250 µM) was prepared in toluene in a 2 mm cuvette under an N_2_ atmosphere. The absorption spectrum of the mixture (bottom, Figure 1a) corresponds to the superposition of the individual chromophores, indicating no significant ground‐state interaction. Upon excitation into the Q bands of **PdOEP** with green light, the TTA‐UC solution produced intense blue emission, confirming successful upconversion.

**FIGURE 1:**
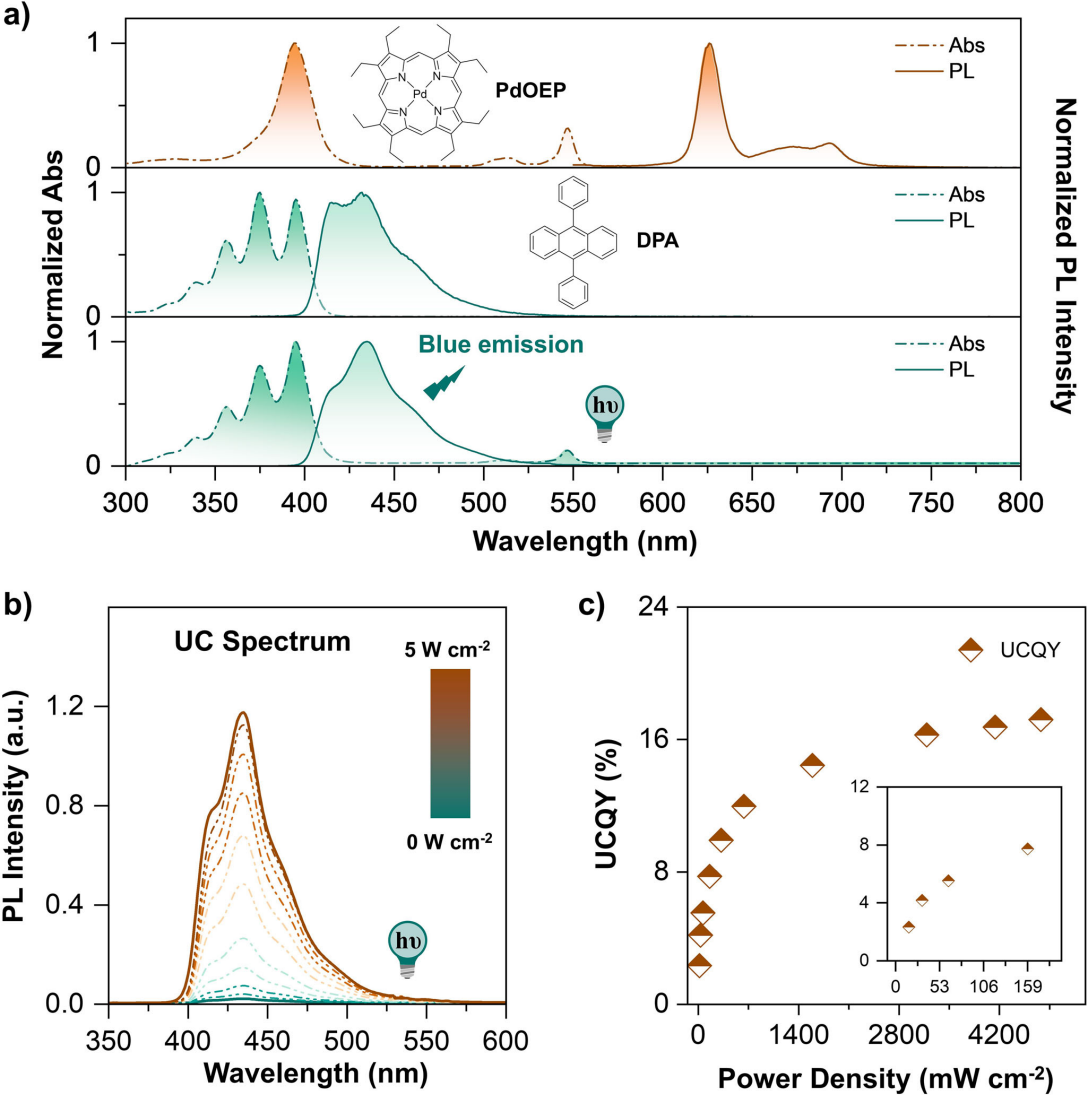
(a) Normalized absorption (Abs, dashed lines) and emission (PL, solid lines) spectra of **PdOEP**, **DPA,** and their TTA‐UC mixture in toluene. (b) Upconversion (UC) emission spectra with varying excitation power densities. (c) UCQY of **PdOEP** and **DPA** in deaerated toluene at various excitation light intensities of the 532 nm laser; the inset depicts the data within the first 200 mW cm^−2^.

The TTA‐UC performance of the **PdOEP**/**DPA** system was further investigated by increasing the green light excitation intensity. For instance, a power‐adjustable 532 nm laser, resulted in a gradual enhancement of the blue emission as shown in Figure 1b. A power‐dependent study demonstrated a transition from a quadratic to a linear regime (for detailed information, see Supporting Information (SI), Section 1, Figure S23), which is a key characteristic of TTA‐UC system [54]. Moreover, efficient triplet energy transfer from **PdOEP** to **DPA** during the TTA‐UC process was then confirmed by steady‐state emission and nanosecond transient absorption (nsTA) spectroscopies (Figures S24–S27), and the UCQY of this TTA‐UC system was determined to be up to 17.2% (out of a 50% maximum, Figure 1c, for detailed information, see Supporting Information, Section 1). Notably, even under subsolar irradiation intensity, such as at 50 mW cm^−2^, the green‐to‐blue UCQY still remains 5.3%, which is much higher than the previously reported UCNPs assisted photoclick reaction that operate under coherent 980 nm laser excitation [29, 55].

The observed efficient blue‐light generation in the TTA‐UC system prompted us to investigate whether a **PQ‐ERA** photoclick reaction could be driven under noncoherent green‐light irradiation. As a model transformation, we employed our previously reported **PQ‐ERA** photoclick reaction consisting of **PQ** and *N*‐Boc‐2,3‐dihydro‐1*H*‐pyrrole (**PY**) molecules [30]. The detailed synthesis of **PQ**s is provided in the Supporting Information (Section 2, Schemes S2, S3, Section 3, Figures S1–S20), while **PY**s were used as commercially obtained without further purification. Under an N_2_ atmosphere, the TTA‐UC solution was mixed directly with **PQ**s and **PY**s (50 and 500 µM, respectively) in toluene within a 10 mm cuvette. Absorption spectra of the mixture revealed characteristic bands attributable to **PdOEP**, **DPA**, and the photoclick reactants (Figure S28), with no evidence of significant ground‐state interactions among the components. Photoreactions were carried out using a green LED equipped with a long‐pass filter (>550 nm, 50 mW cm^−2^) as depicted in Figure 2a (physical setup in Figure S29). Upon green light excitation, the blue upconverted emission generated via TTA‐UC partially overlapped with the absorption band of **PQ** (Figure 2b), thereby enabling the photoclick reaction between **PQ**s and **PY**s. The nsTA measurements of the upconversion photoluminescence (UCPL) lifetime, performed before and after adding **PQ**s (50 µM), are presented in Figure 2c. The rise and decay components (3.1 and 30.3 µs, respectively) remained unchanged upon introduction of **PQ**s. As shown in Figure S30, increasing the **PQ** concentration from 0 to 200 µM only slightly changes the UCPL lifetime. However, increasing the **PQ**s concentration led to a pronounced decrease in UCPL intensity (Figure S31a, b), and the steady‐state Stern–Volmer plot of PL intensity quenching showed a pronounced upward curvature at elevated concentrations (Figure S31c), matching the exponential attenuation predicted by the Beer–Lambert Law for radiative reabsorption. These findings indicate that the attenuation of UCPL arises primarily from reabsorption of the blue photons by **PQ**s rather than FRET.

The **PQ‐PY** reaction under TTA‐UC condition was subsequently evaluated (Figure 3a). The chosen sensitizer/annihilator concentration ratio represents an optimized compromise that maximizes both UCQY and reaction rate (see Figure S32 for the full structure–activity relationship). As a benchmark, the reaction between **PQ**s and **PY**s was first conducted under direct 365 nm UV irradiation in the absence of the TTA‐UC system (Figure S33a). A distinct color change from yellow to colorless was observed in the solution (Figure S34), and UV–visible absorption spectroscopy revealed the decay of the characteristic absorption band at 410 nm and the concomitant formation of a new band centered at 314 nm (Figure S33a). This spectral evolution conclusively confirms the successful photocycloaddition reaction [29, 30, 31, 32]. When the mixture included the TTA‐UC molecules and was irradiated with green light (>550 nm, 50 mW cm^−2^), a similar spectral evolution was observed in UV–visible absorption spectra (Figure 3b), along with a typical color change, matching the photoclick behavior under direct UV excitation. This indicates that the upconverted blue photons from the TTA‐UC process are absorbed by **PQ**, initiating a [4+2] cycloaddition with **PY**, which was also confirmed by ^1^H‐NMR analysis (Figure S9). Complete conversion of **PQ**s was achieved within 5 min under 550 nm LED irradiation. The pseudo‐first‐order kinetics (*k_obs_*), monitored by the absorbance change at 430 nm (Figure 3c), yield a rate constant of 0.007 s^−1^. It achieves comparable kinetics while enabling the activation of **PQ**s with higher triplet energies. These features render the TTA‐UC‐assisted **PQ‐ERA** system highly competitive among existing green‐light‐activated photoclick reactions [32]. Control experiments performed in the absence of either the sensitizer or the annihilator showed no spectral changes under 550 nm LED irradiation (Figure S35), which confirms that the reaction is driven solely by upconverted photons rather than direct excitation or sensitization. These findings further confirmed the stability of both the photoreactants and the upconversion system under the employed reaction conditions, with no detectable degradation or parasitic photoreactivity observed. Experiments under air‐saturated conditions were further tested (Figure S36). As expected, neither upconverted blue emission from **DPA** nor the desired photoclick product of **PQ** was observed in the presence of dissolved oxygen. This null result is fully consistent with efficient triplet quenching by molecular oxygen, which intercepts the **PdOEP** triplet state and completely suppresses both triplet energy transfer to **DPA** and subsequent TTA‐UC. To evaluate whether the **PQ‐ERA** cycloadducts could quench the TTA‐UC process, the calculation of excited‐state energies of the products at the MN15/def2‐TZVP level (SMD, toluene) was performed. The calculated triplet energy (T_1_) of the **PQ_PY** adduct is 2.80 eV, which is substantially higher than that of the sensitizer **PdOEP** (1.87 eV); thus, exothermic TTET from **PdOEP** to the product is thermodynamically forbidden. The singlet state (S_1_) of the product lies at 3.95 eV, with an absorption onset below ∼320 nm that has negligible spectral overlap with the **DPA** upconverted emission (centered around 430–450 nm). Consequently, neither triplet quenching of the sensitizer nor singlet quenching of the annihilator by the cycloadduct occurs, confirming that the UC process remains kinetically unperturbed as the photoclick reaction proceeds to completion.

**FIGURE 2:**
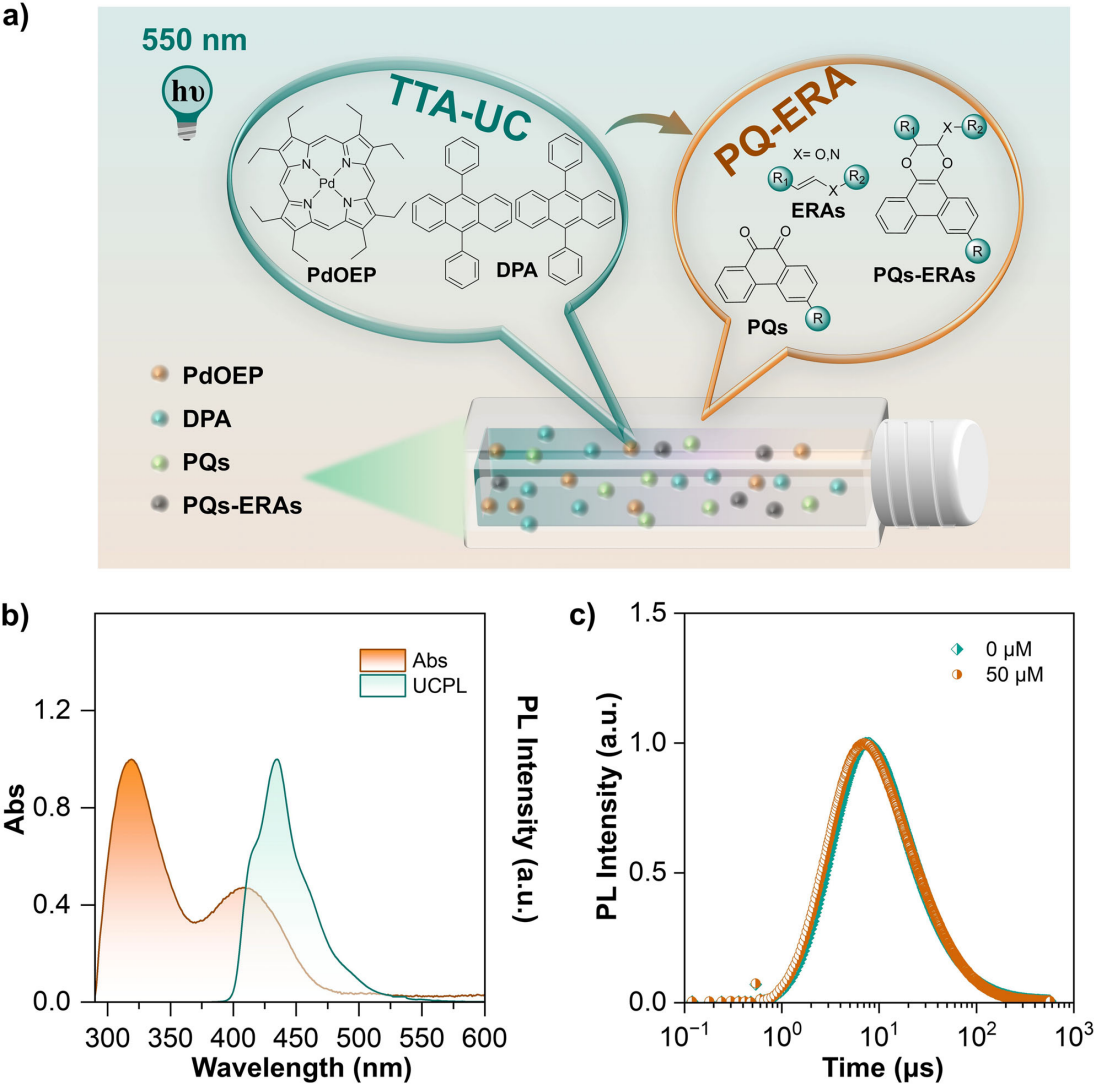
(a) Schematic diagram of TTA‐UC driven photoclick reaction; (b) Overlap of the emission spectra of UCPL and absorption spectra of **PQ;** (c) UCPL lifetime of TTA‐UC sample under 550 nm excitation in toluene before and after addition of **PQ**s (50 µM).

**FIGURE 3:**
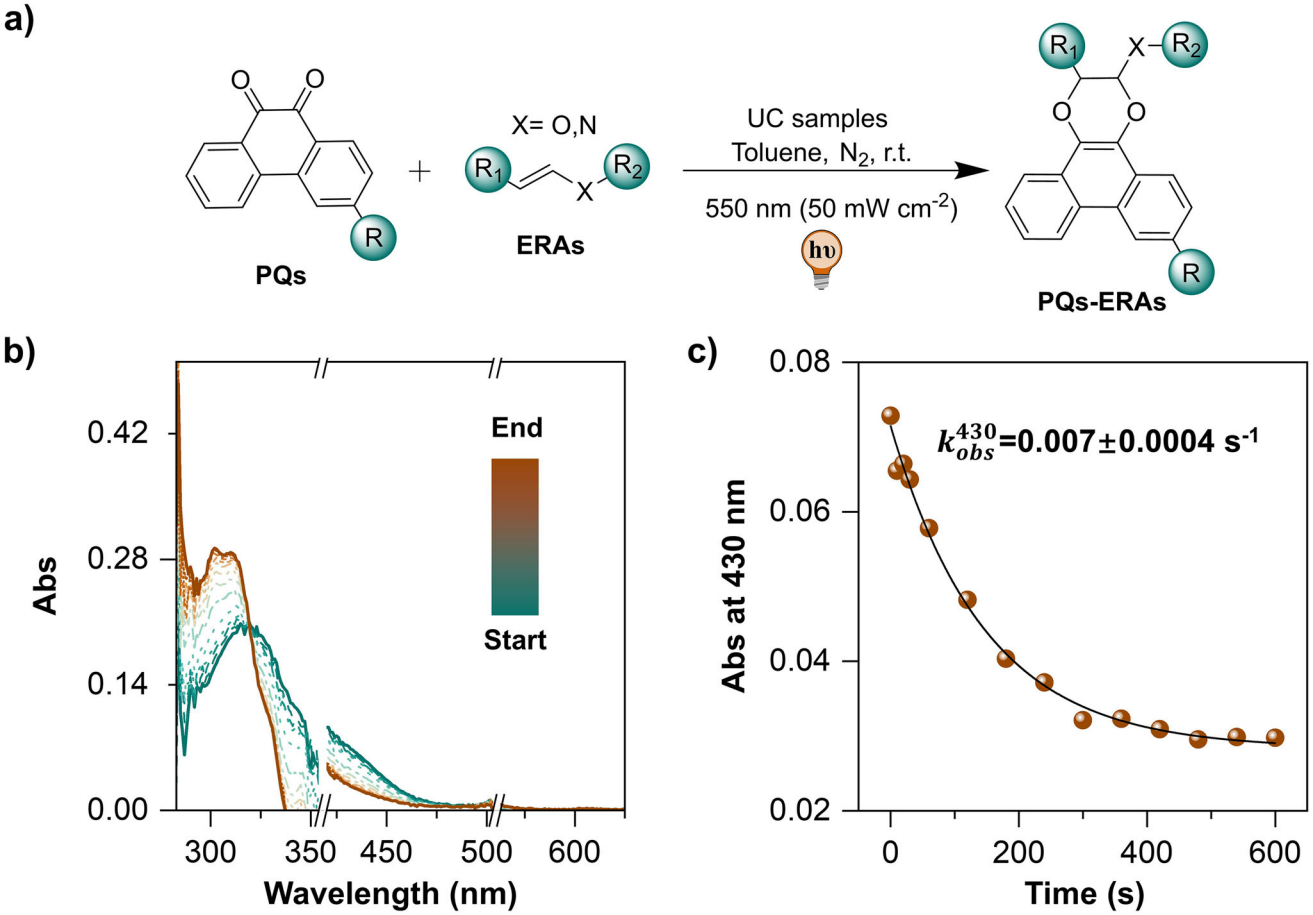
**PQ‐ERA**s photocycloadditions under 550 nm green light irradiation when mixed with TTA‐UC solution. (a) Reaction scheme of **PQ_PY** assisted by TTA‐UC process upon 550 nm LED irradiation; (b) UV–visible absorption spectra of the **PQ_PY** (50 µM **PQ** and 500 µM **PY**) irradiated by 550 nm LED under N_2_ atmosphere; (c) Kinetic traces for photocycloaddition between **PQ_PY** and TTA‐UC sample. *Note*: In (b), dashed break marks are introduced in the wavelength axis at 350–420 and 500–560 nm. The former corresponds to the strong Soret‐band absorption of **PdOEP**, while the latter results from a low signal‐to‐noise ratio caused by minor sampling variations during spectral acquisition.

Having established the efficient green‐light activation of the model **PQ** reaction, the generality of the TTA‐UC approach was investigated using **PQ** derivatives bearing substituents with diverse electronic properties. Three derivatives were examined: the electron‐donating 3‐(thiophen‐2‐yl) phenanthrene‐9,10‐dione (**PQ‐3TP**), the methoxy‐substituted analogue (**PQ‐3OCH_3_**), and the electron‐withdrawing benzotrifluoride‐substituted derivative (**PQ‐3CF_3_**). The synthesis of **PQ** derivatives is detailed in Section 2 of the Supporting Information, and the occurrence of the photoclick reactions under 365 nm UV irradiation were confirmed by UV–visible absorption spectroscopy (Figure S33b–d). These molecules were then separately mixed with **PY** and the TTA‐UC solution under conditions identical to those of the parent **PQ**s to evaluate the scope of the green light activated **PQ‐ERA** cycloaddition.

Reaction progress upon 550 nm LED irradiation was monitored by UV–visible absorption spectroscopy. **PQ‐3TP** exhibited rapid growth of an absorption band at 340 nm in the presence of the TTA‐UC system (Figure 4a). This spectral change indicated fast formation of the photocycloadduct, which was also confirmed by ^1^H‐NMR analysis (Figure S12). The determined rate constant (*k_obs_* = 0.014 s^−1^) confirmed efficient activation via the TTA‐UC pathway. PQ‐3TP with another alkene substrate, 2,3‐dihydrofuran (**DHF**), also underwent the **PQ‐ERA** process in the **PdOEP**/**DPA** TTA‐UC solution, and the rate constant was determined to be 0.005 s^−1^ (Figure S37). Notably, the required excitation intensity in our approach is 160‐fold lower than that of our recently reported NIR‐driven photoclick system employing UCNPs [29] (<50 mW cm^−2^ vs. >8 000 mW cm^−2^), and the reaction time is accelerated by over 10‐fold. To demonstrate the unique advantages of the TTA‐UC system, we also attempted to drive the **PQ‐ERA** reaction using the **PdOEP** alone and UCNPs under the same excitation conditions. Owing to mismatched triplet energy levels between sensitizers and **PQ‐3TP** and the low‐intensity incoherent light source, neither approach produced detectable product (Figures S38, S39). Similarly, green light irradiation of **PQ‐3OCH_3_** system resulted in a decrease of its 390 nm absorption band and the concomitant growth of a new band between 305 and 355 nm, reaching a photostationary state within 5 min (Figure 4b). Comparable spectral evolution was observed for **PQ‐3CF_3_** (Figure 4c). Rate constants derived from absorption changes at 430 nm were 0.007 and 0.015 s^−1^ for **PQ‐3OCH_3_** and **PQ‐3CF_3_**_,_ respectively (Figure 4d). The formation of the [4+2] photocycloaddition product was also confirmed by ^1^H‐NMR (Figures S16, S19).

**FIGURE 4:**
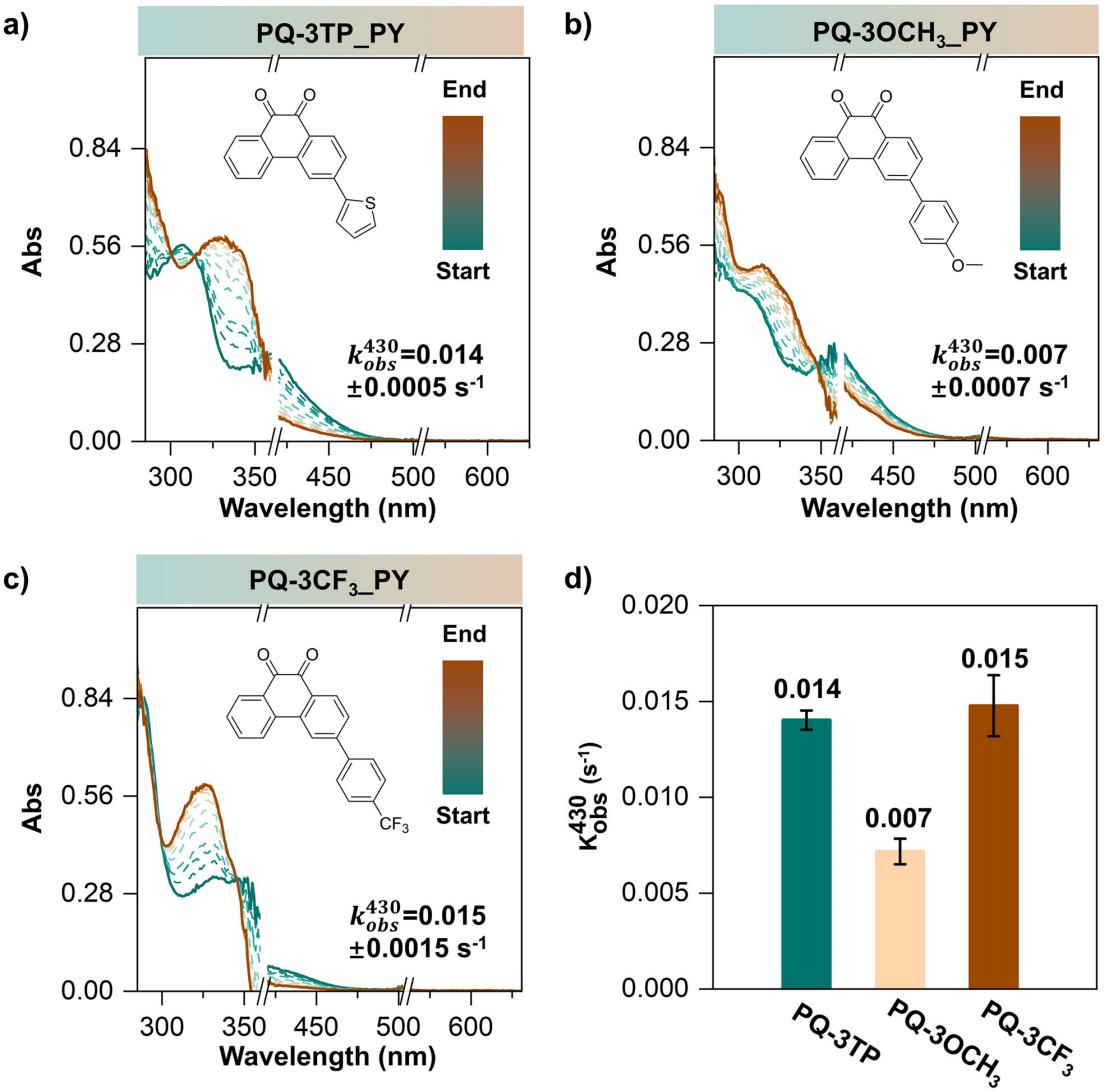
Kinetic analysis of **PQ‐ERA**s photocycloadditions. Time‐resolved UV–visible absorption spectra of (a) **PQ‐3TP**, (b) **PQ‐3OCH_3,_** and (c) **PQ‐3CF_3_** and **PY** mixed with TTA‐UC solution irradiated with a 550 nm LED under N_2_ atmosphere, concentration: 50 µM **PQ**s and 500 µM **PY**; (d) Overview of the first order reaction rate (*k_obs_*) of the **PQ‐ERA**s photoclick reaction system following the absorbance changes at 430 nm. *Note*: In (a–c), the wavelength axis is interrupted by axis break marks in two regions (350–420 and 500–560 nm), as in Figure 3b.

Inspired by these results, we introduced another green‐to‐blue TTA‐UC pair based on an iodinated **BODIPY** sensitizer and perylene annihilator (see Scheme S5 for the TTA‐UC mechanism, Figure S40a, b for the absorption and emission spectra). The detailed synthesis of **BODIPY** is provided in the Supporting Information (Section 2, Scheme S4, Section 3, Figures S21, S22). A mixture of **BODIPY** (4 µM) and perylene (320 µM) in toluene was prepared in a 10 mm cuvette under an N_2_ atmosphere. Upon green‐light excitation of **BODIPY**, the TTA‐UC solution produced intense blue emission in the range 440–520 nm (Figure S40c) with a UCQY of 5.7%. Then, the model reaction of **PQ‐3TP** and **PY** was mixed with **BODIPY**/perylene TTA‐UC solution and irradiated under a low‐intensity green LED. The same characteristic UV–visible spectral changes were observed with a rate constant *k_obs _* = 0.0013 s^−1^ (Figure S41), indicating the successful activation of the photoclick reaction assisted by the **BODIPY**/perylene TTA‐UC system. The slower rate, relative to the **PdOEP**/**DPA** system, is attributed to the red‐shifted perylene emission (>440 nm) and partial reabsorption of its first emission peak, which reduces spectral overlap with the **PQ** absorption (Figure S40d). Nevertheless, it indicates that the TTA‐UC‐assisted photoclick reaction is a general strategy and can be activated as long as the annihilator emission overlaps with the reactant absorption spectrum.

The above results demonstrate the general applicability of the TTA‐UC approach for mediating **PQ‐ERA** reactions across derivatives with varying electronic characters. To assess potential practical utility, a spatially separated configuration was also implemented (Figure S29b). In this setup, the TTA‐UC system was photoexcited in a distinct compartment, with its upconverted emission optically directed toward the physically separated **PQ_PY** mixture (Figure S42). Although this arrangement resulted in a slower reaction rate compared to the directly mixed system, it confirms the feasibility of the approach for scenarios requiring component separation, such as in solid‐state or encapsulated formats, underscoring the robustness and adaptability of the methodology under subsolar irradiance [56, 57].

Encouraged by the efficient organic‐phase photoclick reactivity, we further explored the TTA‐UC system in aqueous media as a step toward potential biological applications. To this end, **PdOEP**, **DPA**, **PQ**s, and **PY** were co‐encapsulated with polyethylene glycol (**PEG**) and redispersed in deionized water, where green‐light‐driven photoclick conversion was successfully observed (see Supporting Information for preparation details and spectral data; Figure S43). Furthermore, the **PQ‐PY** reaction also proceeded in buffers of different pH values (pH 6 and 9, Figure S44). We also demonstrated that the **PQ‐ERA** reaction can be carried out in a hydrogel matrix under low‐power green LED irradiation (*k_obs_* = 0.018 s^−1^). These aqueous‐phase demonstrations confirm that the present TTA‐UC photoclick strategy is compatible with diverse environments, highlighting the potential for future biological and materials applications.

## 3 Conclusion

In summary, we present for the first time an efficient and general strategy for achieving **PQ‐ERA** photoclick reactions via the TTA‐UC process under low‐intensity green light irradiation. Two TTA‐UC systems, comprising **PdOEP**/**DPA** or **BODIPY**/perylene, were introduced and directly combined with the photoclick reaction substrates. The generated UV/blue photons resulting from the TTA‐UC process spectrally overlap with the absorption profile of **PQ** derivatives, thereby facilitating fast and highly efficient photoclick reactions using a low‐intensity 550 nm LED light source (50 mW cm^−2^). The photoclick reaction was observed to be completed within 5 min with a reaction rate constant up to 0.015 s^−1^. Importantly, our strategy demonstrates generality across alkene substrates and **PQ** derivatives bearing electronically diverse substituents, confirming its applicability beyond the parent **PQ‐ERA** process through deliberate structural modification. In addition, the aqueous‐phase green‐light‐induced photoclick reaction was achieved through **PEG** encapsulation, and the system remains active under various conditions, including different pH values and hydrogel matrix, validating its promise for biocompatible photochemical applications. We envision that TTA‐UC‐assisted photoclick reactions will extend well beyond the model systems presented here, offering promising opportunities in areas such as polymer formation, late‐stage functionalization, photopatterning, photoswitching, and photochemical crosslinking.

## Author Contributions

**Qingxin Luan**: investigation, formal analysis, writing – original draft. **Mei Sang**: supervision. **Yuxin He**: investigation. **Hao Yang**: investigation. **Anna M. Doze**: formal analysis. **Lili Hou**: conceptualization, supervision, funding acquisition, writing – review and editing. **Youxin Fu**: conceptualization, supervision, funding acquisition, writing – review and editing. **Ben L. Feringa**: conceptualization, supervision, funding acquisition, writing – review and editing.

## Conflicts of Interest

The authors declare no conflicts of interest.

## Supporting information



The authors have cited additional references within the Supporting Information [58, 59, 60, 61, 62, 63]. **Supporting File**: anie75033‐sup‐0001‐SuppMat.docx.

## Data Availability

The data that support the findings of this study are available in the supplementary material of this article.
