# Supplementary material for: Efficient Green Light‐Induced Photoclick Reactions Enabled by Triplet–Triplet Annihilation Photon Upconversion
Source: Angew Chem Int Ed Engl. 2026 Sep 29;65(46):e8317220. doi: 10.1002/anie.8317220 (PMC13749363; doi:10.1002/anie.8317220)
Supplement: Supplementary file 1 [file ANIE-65-e8317220-s001.docx]

Supporting information for

Efficient Green Light-induced Photoclick Reactions Enabled by Triplet-Triplet Annihilation Photon Upconversion

Qingxin Luan,^[a,b]^ Mei Sang,^[b]^ Yuxin He,^[b]^ Hao Yang,^[b]^ Anna M. Doze,^[c]^ Lili Hou,*^[b]^ Youxin Fu,*^[a]^ Ben L. Feringa*^[b,c]^

^[a]^ College of Science, Nanjing Forestry University, Nanjing 210037, China

^[b]^ State Key Laboratory of Precision Measurement Technology and Instruments, School of Precision Instruments and Optoelectronics Engineering, Tianjin University, 92 Weijin Road, Tianjin, 300072, China

^[c]^ Centre for Systems Chemistry, Stratingh Institute for Chemistry, Faculty for Science and Engineering, University of Groningen, Nijenborgh 4, 9747AG Groningen, The Netherlands.

Table of Contents

[1.	General Information	3](#_Toc238466236)

[1.1 Chemicals	3](#_Toc238466237)

[1.2 Steady-state absorption and emission spectra	3](#_Toc238466238)

[1.3 TTA-UC measurements	3](#_Toc238466239)

[1.4 Nanosecond transient absorption spectroscopy (nsTA)	3](#_Toc238466240)

[1.5 Reaction monitoring	4](#_Toc238466241)

[1.6 Analysis	5](#_Toc238466242)

[1.7 Kinetics fitting	5](#_Toc238466243)

[1.8 DFT calculation	6](#_Toc238466244)

[1.9 Preparation of PEG-encapsulated reaction system for aqueous photoclick reaction	7](#_Toc238466245)

[1.10 Preparation of pH buffers and hydrogel for photoclick reaction	7](#_Toc238466246)

[2.	Synthetic procedures	7](#_Toc238466247)

[2.1 Synthesis of PQ-3TP	7](#_Toc238466248)

[2.2 Synthesis of PQ-3CF_3_	8](#_Toc238466249)

[2.3 Synthesis of PQ-3OCH_3_	8](#_Toc238466250)

[2.4 Synthesis of PQ-PY	9](#_Toc238466251)

[2.5 Synthesis of PQ-3TP-PY	9](#_Toc238466252)

[2.6 Synthesis of PQ-3CF_3_-PY	9](#_Toc238466253)

[2.7 Synthesis of PQ-3OCH_3_-PY	10](#_Toc238466254)

[2.8 Synthesis of core-shell-shell UCNP (NaYF_4_ @NaYF_4_:75%Yb^3+^,0.5%Er^3+^@NaYF_4_)	10](#_Toc238466255)

[2.9 Synthesis of BODIPY	11](#_Toc238466256)

[3.	NMR spectra	12](#_Toc238466257)

[4.	Photophysical and Photochemical Studies by UV-Visible Spectroscopy, Emission Spectroscopy, nsTA Spectroscopy	23](#_Toc238466258)

[Reference	37](#_Toc238466259)

1. General Information
   1.1 Chemicals

Unless noted otherwise, all chemicals were obtained commercially in high purity and used as received. (Palladium(II) octaethylporphyrin (**PdOEP**), PorphyChem; 9,10-diphenylanthracene (**DPA**), 98%, Zancheng (Tianjin) Technology Co., Ltd.; perylene, 98%, Meryer (Shanghai) Biochemical Technology Co., Ltd.; 9,10-Phenanthraquinone (**PQ**), 98%, Heowns Biochem Technologies, LLC, Tianjin; N-boc-2,3-dihydro-1H-pyrrole (**PY**), 95%, LeYan; 2,3-dihydrofuran (**DHF**), 98%, Heowns Biochem Technologies, LLC, Tianjin; Rhodamine 6G (**R-6G**), 95%, Heowns Biochem Technologies, LLC, Tianjin; 1,2-distearoyl-sn-glycero-3-phosphoethanolamine-N-[methoxy(polyethylene glycol)-2000] (**PEG**) (ammonium salt, 99.48%), Pusenruite (Tianjin) Biomedical Technology Co., Ltd.; hydrogel, Heowns Biochem Technologies, LLC, Tianjin; Toluene (TOL), 99.5%, Tianjin Yuanli Chemical Co., Ltd.; Ethanol, 99.7%, Tianjin Aopu Sheng Chemical Co., Ltd.; Solvents used were of analytical grade. All other chemicals were used as received unless otherwise indicated. Deionized water was used throughout. All oxygen or moisture-sensitive reactions were performed in dried glassware and under N_2_ atmosphere using standard Schlenk techniques. Dry solvents were obtained from an MBraun solvent purification system (SPS). Column chromatography was performed on silica gel (Silica 60 M, 0.04-0.063 mm).

1.2 Steady-state absorption and emission spectra

Steady-state absorption spectra were recorded on an Agilent-Cary 60 UV−Vis spectrophotometer. Fluorescence spectra were carried out on Agilent Cary Eclipse Fluorescence spectrofluorometer (G9800A).

1.3 TTA-UC measurements

Steady-state upconversion photoluminescence (UCPL) measurements were performed on a home-built system,^[1]^ consisting of a continuous-wave 532 nm laser (MGL-FN-532-2W, beam diameter 1.172 mm, Changchun New Industries Optoelectronics Technology Co., Ltd.) as the excitation source, a monochromator (Omni-λ3007i, Zolix), and a photomultiplier tube (PMT) detector. In addition, to vary the laser power, an adjustable light attenuator was used to adjust the laser power. A data acquisition card was used for signal acquisition, which converts the analog signal output from the PMT into a digital signal, and spectra were recorded using home-built LabVIEW software.

Photoclick reaction measurements were carried out in TOL using 10 mm quartz cuvettes; other photophysical measurements were carried out in TOL using 2 mm quartz cuvettes. All samples were prepared in a nitrogen-filled glovebox (Mikrouna (Shanghai) Co., Ltd.) with ≤ 0.1 ppm oxygen levels, and sealed with air-tight cap screws and parafilm before taking out from the glovebox.

Fluorescence quantum yields of the annihilators were determined using relative method, and **R-6G** in deoxygenated ethanol (Ф_F_= 0.94)^[2]^ as the reference compound.

Technically, the UCQY ($\Phi_{UC}$) of a triplet-triplet annihilation upconversion (TTA-UC) sample can be determined by using a relative quantum yield method, which was calculated using equation (S1),^[3-4]^

$$\begin{aligned} \Phi_{uc}=\Phi_{r}\frac{I_{uc}}{I_{r}}\frac{A_{r}}{A_{uc}}\frac{n_{uc}^{2}}{n_{r}^{2}}\#(S1) \end{aligned}$$

where, $uc, r$ represent as TTA-UC sample and reference sample, respectively, $\Phi$ is photoluminescence quantum yield, $A$ is absorbance at the excitation wavelength, $I$ is the integral emission intensity, $n$ represents solvent refractive index.

1.4 Nanosecond transient absorption spectroscopy (nsTA)

Nanosecond transient spectrum measurements were performed on a Transient Absorption Spectrometer (LP980, Edinburgh Instruments) using an adjustable YAG laser (Agilite, Amplitude Continuum) equipped with an OPO (Horizon, Amplitude Continuum) generating a 10 ns pump beam. A 150 W ozone-free, xenon arc lamp was used as the probe light. The time-resolved decays were measured on a 5-stage PMT (R13456P, Hamamatsu) coupled with a monochromator (TMS302, Edinburgh Instruments) and recorded on an oscilloscope (RTM3002, Rohde and Schwarz). The transient spectra were recorded on a gated ICCD camera from Andor (DH320T-25F-03, Oxford Instruments).

1.5 Reaction monitoring

Reaction solutions of **PdOEP** (30 μM), **DPA** (250 μM), **PQ**s (50 μM) and **PY** (500 μM) in TOL were prepared. The photoclick reactions were monitored by a UV-Visible (UV-vis) absorption spectrophotometer (Agilent-Cary 60) under 550 nm LED excitation coupled with a long-pass filter (>550 nm). For the directly mixed system, the sample was placed in a cuvette with a transparent bottom and stirred with a magnetic stir bar (Figure S29a). For the physically separated system, the upconversion mixture was confined in an inner tube inserted into the cuvette containing the reaction substrates (Figure S29b). All the kinetic data presented as the mean ± standard deviation from at least three independent replicate experiments (n = 3).

The UCQY and photoclick rate were measured as a function of the sensitizer/annihilator ratio (Figure S32). The photoclick rate peaked at a ratio of ~0.12 (30:250 μM), whereas the UCQY of the **PdOEP**/**DPA** system was also approaching its maximum. However, further increasing the **PdOEP** ratio only slightly improves the UCQY while significantly decreasing the photoclick rate. This can be attributed to that excess sensitizers introduce strong reabsorption of upconverted emission, reducing the photon flux available for photoclick reactions. This marked decoupling reveals a transition from photon-limited to photon-utilization-limited kinetics in the TTA-UC-assisted photoclick reaction.

**Table S1.** Key performance parameters of representative TTET, UCNP, and TTA-UC systems for the **PQ-ERA** photoclick reaction.

| **Category** | **Excitation wavelength**  **(light source)** | **Power density** | **Reaction efficiency** | **Substrate scope & generality** | **Key limitations** | **Ref** |
| --- | --- | --- | --- | --- | --- | --- |
| TTET sensitization | 530 nm (green LED) | ~ mW·cm^-2^ (incoherent, continuous-wave (CW)) | Medium  (time: 0-10 min) | Low | Sensitizer absorption; energy level matching; oxygen-sensitive | ^[5]^ |
| UCNP-based (lanthanide nanoparticle) | 980 nm (NIR laser)  808 nm (diode laser) | 8-28 W·cm^-2^ (CW or pulsed laser) | Low  (time: 10- > 60 min) | Medium | High-power coherent laser; low quantum yield of UCNP; thermal effects; poor reproducibility of nanoparticle batches; UCNP dispersion, expensive and oxygen-sensitive | ^[6]^ |
| **This work (TTA-UC)** | **≥ 550 nm (green LED)** | **~10-50 mW·cm^-2^ (incoherent, CW)** | **High**  **(time < 5 min)** | **High** | **Oxygen-sensitive** | **-** |

As summarized in Table S1, compared to UCNP-based strategies, which require high-intensity coherent NIR laser excitation (typically 1-10^4^ W·cm^-2^) due to the nonlinear multiphoton process of lanthanide upconversion, our TTA-UC approach uses several orders of magnitude lower power density and eliminates the need for coherent light sources. In contrast to TTET sensitization, which is intrinsically limited by the sensitizer’s absorption window and the requirement for well-matched triplet energy levels between the sensitizer and **PQ** molecules, our approach offers greater flexibility in excitation and energy-transfer design.

1.6 Analysis

Mass spectra were recorded on an AEI-MS-902 mass spectrometer (EI+) or a LTQ Orbitrap XL (ESI+, ESI-, APCI+). ^1^H- and ^13^C NMR were recorded on Bruker AM-400 Spectrometer (400 MHz and 100.59 MHz, respectively using DMSO‑*d_6_* as solvent. Data are reported as follows: chemical shifts, multiplicity (s = singlet, d = doublet, t = triplet, q = quartet, m = multiplet), coupling constants (Hz), and integration.

1.7 Kinetics fitting

The photoluminescence (PL) decay curves of **PdOEP** and exciton bleach (XB), PL decay curves of **PdOEP** and **DPA** complexes were fitted by multiple exponential functions. The average lifetime $\tau_{ave}$ was calculated according to eq. S2,

$$\begin{aligned} \tau_{ave}=\frac{\sum_{i} A_{i}\tau_{i}}{\sum_{i} A_{i}}\#\left( S2 \right) \end{aligned}$$

Where$A_{i}$, $\tau_{i}$ are the amplitude and lifetime, respectively, of the i-th component.

The photophysics of **PdOEP** is well established and is illustrated in the Jablonski diagram (Scheme S1).

**Scheme S1.** Jablonski diagram of **PdOEP**. Ex: Excitation; Fluo: fluorescence; Phos: phosphorescence.

Following excitation, the excited singlet state decays with an overall rate constant:

$$\begin{aligned} k_{S}=k_{f}+k_{isc}\#\left（ S3 \right） \end{aligned}$$

Where $k_{f}$ is the fluorescence rate constant and $k_{isc}$ is the intersystem crossing rate constant. For **PdOEP**, the heavy-atom effect ensures $k_{isc}\gg k_{f}$ (typically $k_{isc}\sim{10}^{11}-{10}^{12}\text{ }\text{s}^{-1}$ and $k_{f}\sim{10}^{6}-{10}^{7}\text{ }\text{s}^{-1}$), giving a near-unity triplet quantum yield and a singlet lifetime on the order of picoseconds or less. Consequently, on the nanosecond-to-microsecond time scale accessible to our apparatus, only the triplet state is observed. the recovery of the XB directly reflects the repopulation of the S_0_ state as the T_1_ state decays. Therefore, the XB decay kinetics are governed exclusively by the triplet lifetime.

The triplet state T_1_ decays to S_0_ with a rate constant:

$$\begin{aligned} k_{T}=k_{r}+k_{nr} \#\left（ S4 \right） \end{aligned}$$

Where $k_{r}$ is the radiative (phosphorescence; $k_{r}=1/\tau_{E_{m}}$) rate constant and $k_{nr}$ is the sum of all non-radiative decay rate constants. The intrinsic triplet lifetime is $k_{T}=1/\tau_{T}$. Both the ground-state bleach recovery and the triplet lifetime directly monitor the T_1_ population, and therefore must yield the same lifetime provided that no other absorbing species interfere. The $k_{r}$ is a component of $k_{T}$, so the lifetime of $\tau_{E_{m}}$ is longer than the triplet decay lifetime.

Figure S24 shows a reduction in the **PdOEP** triplet lifetime (670 nm) from 40.1 μs to 2.3 μs in the presence of **DPA**, and XB (545 nm) exhibits similar kinetic characteristics (Figure S25). The intrinsic phosphorescence decay (Figure S26) from the T_1_ state to S_0_ monitored at 680 nm, which also shows a reduction in the presence of **DPA** (55.1 μs to 2.4 μs). Furthermore, the nsTA spectra clearly showed the emergence of the **DPA** triplet state absorption at approximately 450 nm (Figure S27), providing the TET occurred in this sample.

**PQ**s**_PY** formation was fitted to an exponential equation S3,

$$\begin{aligned} y={(y}_{0}-a)\cdot e^{k_{obs}\times t}+b\#\left( S3 \right) \end{aligned}$$

Where y represents absorbance, $k_{obs}$ is the pseudo-first-order kinetics, t is the time. a and b are constant value.

1.8 DFT calculation

The density functional theory (DFT) calculations on the **PQ** derivatives and **PQ-ERA** product using Gaussian software. The ground-state geometry was optimized and the excited-state energies were calculated at the MN15/def2-TZVP level of theory with the SMD implicit solvation model (solvent = toluene).

**Table S2.** Calculated singlet and triplet excitation energies (eV) of **PQ** derivatives and the **PQ-ERA** product at the MN15/def2-TZVP level (SMD, toluene).

|  | **S_1_ (eV)** | **T_1_ (eV)** |
| --- | --- | --- |
| **PQ**  **PQ_PY** | 2.30/2.33^a^  3.95 | 2.07/2.13^a^  2.80 |
| **PQ-3TP**  **PQ-3TP_PY** | 2.30  3.78 | 2.08  2.57 |
| **PQ-3OCH_3_**  **PQ-3OCH_3__PY** | 2.31  3.83 | 2.08  2.70 |
| **PQ-3CF_3_**  **PQ-3CF_3__PY** | 2.29  3.86 | 2.07  2.68 |

a. Adiabatic excitation energies (eV) of S_1_ and T_1_ states of **PQ** obtained at the MN15/def2-TZVP/PCM (MeCN) level.^[5]^

1.9 Preparation of PEG-encapsulated reaction system for aqueous photoclick reaction

**PdOEP**, **DPA**, **PQ**s, and **PY** were combined with **PEG** (average Mw 2000) at a total solute-to-**PEG** mass ratio of 1:100 and dissolved in toluene. The mixture was stirred at 30 °C and 850 rpm for 12 h, after which toluene was removed by rotary evaporation. The obtained solids were redissolved in deionized water to give a clear aqueous dispersion. For the photoclick reaction, 2 mL of this dispersion was placed in a 10 × 10 mm cuvette, deoxygenated by nitrogen purging, and irradiated with a 550 nm LED equipped with a high-pass filter. Reaction progress was monitored by UV-vis absorption spectroscopy.

1.10 Preparation of pH buffers and hydrogel for photoclick reaction

Buffers of different pH were prepared by adjusting with HCl and K_2_CO_3_, and the pH was confirmed using pH test strips. The **PEG**-encapsulated mixture dispersed in deionized water was then added. The hydrogel was prepared at a concentration of 200 mg/mL by dissolving the **PEG**-encapsulated mixture in a mixture of deionized water and ethanol (7.5:2.5, v/v) and allowing complete dissolution before use. For the photoclick reaction, 2 mL of the dispersion was placed in a 10 × 10 mm cuvette, deoxygenated by nitrogen purging, and irradiated with a 550 nm LED equipped with a high-pass filter. Reaction progress was monitored by UV-vis absorption spectroscopy.

2. Synthetic procedures

**Scheme S2.** Synthesis of **PQ** derivatives.

2.1 Synthesis of PQ-3TP

A Schlenk tube containing 3-bromophenanthrene-9,10-dione (**PQ-3Br**, 100 mg, 0.35 mmol), and 2-thienylboronic acid (50 mg, 0.38 mmol), and Pd(PPh_3_)_4_ (23 mg, 0.02 mmol) was evacuated and backfilled with N_2_ for three times. After the addition of degassed aq. K_2_CO_3_ solution (150 mg in 1.5 mL H_2_O), ethanol (2 ml), and toluene (8 mL), the tube was sealed under N_2_ atmosphere and heated at 80 ^o^C for 12 h. After being cooled to room temperature, the reaction mixture was diluted with dichloromethane, the phases were separated and the organic layer was washed with water and dried over anhydrous Na_2_SO_4_. Upon removal of solvents in vacuo, the crude product was purified by column chromatography over silica gel eluted with ethyl acetate/dichloromethane (1/5, v/v) to obtain **PQ-3TP** as an orange solid in 55% yield (0.20 mmol, 55 mg). ^1^H NMR (400 MHz, DMSO-*d_6_*) δ 8.52 (d, J = 6.3 Hz, 2H), 8.07 – 8.01 (m, 2H), 8.00 (d, J = 3.5 Hz, 1H), 7.81 (t, J = 7.7 Hz, 1H), 7.78 – 7.74 (m, 2H), 7.56 (t, J = 7.5 Hz, 1H), 7.26 (dd, J = 5.0, 3.7 Hz, 1H). ^13^C NMR (101 MHz, DMSO-*d_6_*) δ 178.8, 142.2, 136.1, 130.9, 129.5, 129.0, 127.7, 126.8, 121.3. HR-MS (ESI) m/z, calculated for [M+H]+: 291.0474; found: 291.0474.

2.2 Synthesis of PQ-3CF_3_

The title compound was synthesized using the same method as for **PQ-3TP**, employing (4-(trifluoromethyl)phenyl)boronic acid (40 mg, 0.21 mmol) to obtain **PQ-3CF_3_** as a yellow solid (42 mg, 0.119 mmol, yield 70%). ^1^H NMR (600 MHz, DMSO-*d_6_*) δ 8.61 (d, J = 1.6 Hz, 1H), 8.57 (d, J = 8.0 Hz, 1H), 8.17 – 8.13 (m, 3H), 8.07 (dd, J = 7.7, 1.4 Hz, 1H), 7.91 (dt, J = 8.1, 1.6 Hz, 3H), 7.84 – 7.80 (m, 1H), 7.60 – 7.56 (m, 1H). ^13^C NMR (151 MHz, DMSO-*d_6_*) δ 179.4, 179.1, 145.5, 143.1, 136.6, 135.8, 135.5, 131.9, 131.3, 130.5, 130.1, 129.6, 128.7, 128.3, 126.3, 125.5, 123.6. ^19^F NMR (376 MHz, DMSO-*d_6_*) δ -60.64. HR-MS (ESI) m/z, calculated for [M+H]+: 353.0784; found: 353.0776.

2.3 Synthesis of PQ-3OCH_3_

The title compound was synthesized using the same method as for **PQ-3CF_3_** employing (4-methoxyphenyl)boronic acid (0.30 mmol, 50 mg) to obtain **PQ-3OCH_3_** as a yellow solid (50 mg, 0.15 mmol, yield 53%). ^1^H NMR (600 MHz, DMSO-*d_6_*) δ 8.56 (d, J = 7.9 Hz, 1H), 8.49 (d, J = 1.6 Hz, 1H), 8.08 (d, J = 8.1 Hz, 1H), 8.05 (dd, J = 7.8, 1.4 Hz, 1H), 7.95 – 7.89 (m, 2H), 7.85 – 7.78 (m, 2H), 7.59 – 7.53 (m, 1H), 7.14 – 7.08 (m, 2H), 3.85 (s, 3H). ^13^C NMR (151 MHz, DMSO-*d_6_*) δ 179.8, 179.1, 160.6, 146.9, 136.4, 135.9, 135.8, 131.8, 131.2, 130.6, 129.9, 129.5, 129.1, 127.3, 125.3, 122.2, 115.0, 55.8. HR-MS (ESI) m/z, calculated for [M+H]+: 315.1016; found: 315.1020.

**Scheme S3.** Synthesis of **PQ**s**-PY**.

2.4 Synthesis of PQ-PY

**Direct irradiation system: PQ** (0.2 mmol, 40 mg) and **PY** (1 mmol, 174 mg) were dissolved in 20 mL MeCN. Then, the reaction mixture was stirred and irradiated using hand-held LED lamp (395 nm, 88 mW cm^-2^) at a fixed distance for 1 min under N_2_ atmosphere. The progress of the reaction was monitored by TLC. After full consumption of **PQ**, the volatiles were evaporated and the resulting residue was purified by column chromatography on silica gel (dichloromethane/ethyl acetate = 1:4, v/v) to afford **PQ-PY** as a colorless powder (54 mg, 0.14 mmol, 75 % yield). ^1^H NMR (400 MHz, DMSO-*d_6_*) δ 8.73 (d, *J* = 8.2 Hz, 2H), 8.05 (t, *J* = 6.6 Hz, 2H), 7.63 (q, *J* = 6.7 Hz, 2H), 7.57 (tt, *J* = 7.0, 3.3 Hz, 2H), 5.65 (s, 1H), 5.13 (ddd, *J* = 10.4, 6.8, 3.9 Hz, 1H), 3.50 (t, *J* = 9.6 Hz, 1H), 2.34 (dt, *J* = 12.2, 7.0 Hz, 1H), 2.02 – 1.90 (m, 1H), 1.48 (s, 9H). ^13^C NMR (101 MHz, DMSO-*d_6_*) δ 153.7, 127.5, 126.7, 126.6, 125.8, 125.7, 123.4, 120.7, 80.5, 28.5. HR-MS (ESI) m/z, calculated for [M+Na]+: 400.1529; found: 400.1526.

**TTA-UC system: PQ** (0.05 mmol, 10 mg) and **PY** (0.25 mmol, 44 mg) were dissolved in 15 mL toluene, with **PdOEP**/**DPA** addition. Then, the reaction mixture was stirred and irradiated using 550 nm LED lamp at a fixed distance for 10 min under N_2_ atmosphere. The progress of the reaction was monitored by TLC. After full consumption of **PQ**, the volatiles were evaporated and the resulting residue was purified by column chromatography on silica gel (dichloromethane/ethyl acetate = 1:4, v/v) to afford **PQ-PY** as a colorless powder (11 mg, 0.03 mmol, 60 % yield). ^1^H NMR (400 MHz, DMSO-*d*_6_) δ 8.78 (d, *J* = 8.1 Hz, 2H), 8.13 – 8.05 (m, 2H), 7.72 – 7.65 (m, 2H), 7.62 (tdd, *J* = 7.0, 3.1, 1.6 Hz, 2H), 5.70 (s, 1H), 5.22 – 5.15 (m, 1H), 3.55 (t, *J* = 10.2 Hz, 1H), 3.49 – 3.40 (m, 1H), 2.43-2.34 (m, 1H), 2.05 – 1.95 (m, 1H), 1.53 (s, 9H). Data is in accordance with the direct irradiation pathway result.

2.5 Synthesis of PQ-3TP-PY

**Direct irradiation system:** The title compound was synthesized using the same method as for **PQ-PY**, employing **PQ-3TP** (0.15 mmol, 44 mg) to afford **PQ-3TP-PY** as a colorless powder (50 mg, 0.11 mmol, 75 % yield). ^1^H NMR (400 MHz, DMSO-*d_6_*) δ 8.96 (s, 1H), 8.90 (d, *J* = 8.2 Hz, 1H), 8.08 (q, *J* = 6.8, 5.2 Hz, 2H), 7.95 – 7.89 (m, 1H), 7.83 – 7.80 (m, 1H), 7.72 – 7.66 (m, 1H), 7.65 – 7.58 (m, 2H), 7.23 – 7.18 (m, 1H), 5.72 – 5.65 (m, 1H), 5.16 (ddd, *J* = 10.4, 6.7, 3.9 Hz, 1H), 3.57 – 3.49 (m, 1H), 3.42 (dd, *J* = 11.1, 5.0 Hz, 1H), 2.37 (dt, *J* = 12.3, 6.9 Hz, 1H), 2.04 – 1.94 (m, 1H), 1.50 (s, 9H). ^13^C NMR (101 MHz, DMSO-*d_6_*) δ 153.6, 143.9, 131.3, 129.1, 127.8, 127.2, 126.5, 126.3, 125.8, 125.3, 124.7, 123.7, 121.7, 120.8, 119.4, 80.6, 28.5. HR-MS (ESI) m/z, calculated for [M+Na]+: 482.1397; found: 482.1395.

**TTA-UC system:** The title compound was synthesized using the same method as for **PQ-PY**, employing **PQ-3TP** (0.05 mmol, 15 mg) to afford **PQ-3TP-PY** as a colorless powder (13 mg, 0.03 mmol, 55 % yield). ^1^H NMR (400 MHz, DMSO-*d*_6_) δ 9.00 (s, 1H), 8.93 (d, *J* = 8.0 Hz, 1H), 8.11 (t, *J* = 7.4 Hz, 2H), 7.98 – 7.92 (m, 1H), 7.86 (d, *J* = 3.0 Hz, 1H), 7.71 (d, *J* = 7.1 Hz, 1H), 7.67 – 7.62 (m, 2H), 7.27 – 7.22 (m, 1H), 5.70 (s, 1H), 5.24 – 5.14 (m, 1H), 3.56 (t, *J* = 9.3 Hz, 1H), 3.47 – 3.41 (m, 1H), 2.40 (dt, *J* = 12.0, 6.4 Hz, 1H), 2.01 (p, *J* = 8.1 Hz, 1H), 1.53 (s, 9H). Data is in accordance with the direct irradiation pathway result.

2.6 Synthesis of PQ-3CF_3_-PY

**Direct irradiation system:** The title compound was synthesized using the same method as for **PQ-PY**, employing **PQ-3CF_3_** (0.14 mmol, 50 mg) to afford **PQ-3CF_3_-PY** as a colorless powder (50 mg, 0.095 mmol, yield 71 %). ^1^H NMR (400 MHz, DMSO-*d_6_*) δ 9.11 – 9.08 (m, 1H), 8.98 (d, *J* = 8.1 Hz, 1H), 8.19 – 8.12 (m, 3H), 8.11 – 8.06 (m, 1H), 8.02 (td, *J* = 8.6, 8.2, 2.4 Hz, 1H), 7.85 (d, *J* = 8.3 Hz, 2H), 7.73 – 7.66 (m, 1H), 7.66 – 7.60 (m, 1H), 5.71 (d, *J* = 12.7 Hz, 1H), 5.18 (ddd, *J* = 10.5, 6.8, 3.9 Hz, 1H), 3.53 (t, *J* = 9.6 Hz, 1H), 3.48 – 3.37 (m, 1H), 2.37 (ddd, *J* = 11.9, 9.3, 7.0 Hz, 1H), 1.99 (p, *J* = 9.5 Hz, 1H), 1.50 (s, 9H), ^13^C NMR (101 MHz, DMSO-*d_6_*) δ 153.6, 144.5, 135.6, 128.3, 127.8, 127.2, 127.1, 126.8, 126.2, 126.1, 125.8, 123.9, 121.9, 121.7, 120.8, 120.2, 112.6, 80.5, 28.5, 27.9. ^19^F NMR (376 MHz, DMSO-*d_6_*) δ -60.82, HR-MS (ESI) m/z, calculated for [M+H]+: 522.1842; found: 522.1875.

**TTA-UC system:** The title compound was synthesized using the same method as for **PQ-PY**, employing **PQ-3CF_3_** (0.06 mmol, 21 mg) to afford **PQ-3CF_3_-PY** as a colorless powder (19 mg, 0.037 mmol, yield 62 %). ^1^H NMR (400 MHz, *CDCl_3_*) δ 8.79 (s, 1H), 8.67 (d, *J* = 7.7 Hz, 1H), 8.33 – 8.16 (m, 2H), 7.84 (dd, *J* = 13.6, 8.5 Hz, 3H), 7.75 (d, *J* = 8.1 Hz, 2H), 7.61 (dt, *J* = 15.2, 6.8 Hz, 2H), 5.64 (s, 1H), 5.01 (s, 1H), 3.81 – 3.65 (m, 1H), 3.53 (q, *J* = 9.9 Hz, 1H), 2.40 (dt, *J* = 13.8, 7.1 Hz, 1H), 2.28 – 2.11 (m, 1H), 1.60 (s, 9H). Data is in accordance with the direct irradiation pathway result.

2.7 Synthesis of PQ-3OCH_3_-PY

**Direct irradiation system:** The title compound was synthesized using the same method as for **PQ-PY** using **PQ-3OCH_3_** (40 mg, 0.13 mmol) to obtain **PQ-3OCH_3_-PY** as colourless powder (40 mg, 0.08 mmol, yield 64%). ^1^H NMR (400 MHz, DMSO-*d_6_*) δ 8.94 (d, *J* = 7.3 Hz, 2H), 8.09 (dd, *J* = 16.3, 7.7 Hz, 2H), 7.97 – 7.90 (m, 1H), 7.86 (d, *J* = 8.8 Hz, 2H), 7.69 – 7.63 (m, 1H), 7.60 (td, *J* = 6.9, 2.2 Hz, 1H), 7.07 (d, *J* = 8.8 Hz, 2H), 5.70 (d, *J* = 10.5 Hz, 1H), 5.17 (ddd, *J* = 10.5, 6.8, 4.2 Hz, 1H), 3.82 (s, 3H), 3.53 (t, *J* = 9.6 Hz, 1H), 3.47 – 3.37 (m, 1H), 2.42 – 2.33 (m, 1H), 1.99 (p, *J* = 10.2, 9.7 Hz, 1H), 1.50 (s, 9H). ^13^C NMR (151 MHz, DMSO-*d_6_*) δ = 159.5, 153.7, 137.1, 132.9, 128.7, 127.6, 127.3, 126.9, 126.8, 126.0, 125.7, 123.9, 121.5, 120.8, 120.4, 114.9, 80.6, 55.7, 28.5. HR-MS (ESI) m/z, calculated for [M+Na]+: 506.1938; found: 506.1922.

**TTA-UC system:** The title compound was synthesized using the same method as for **PQ-PY** using **PQ-3OCH_3_** (16 mg, 0.05 mmol) to obtain **PQ-3OCH_3_-PY** as colourless powder (14 mg, 0.03 mmol, yield 60%). ^1^H NMR (600 MHz, *CDCl_3_*) δ 8.78 (s, 1H), 8.71 (d, *J* = 7.9 Hz, 1H), 8.22 (td, *J* = 18.2, 17.5, 8.0 Hz, 2H), 7.89 – 7.82 (m, 1H), 7.74 (d, *J* = 8.4 Hz, 2H), 7.68 – 7.62 (m, 1H), 7.60 (t, *J* = 6.9 Hz, 1H), 7.08 (d, *J* = 8.6 Hz, 2H), 5.65 (s, 1H), 5.04 (s, 1H), 3.92 (s, 3H), 3.74 (dd, *J* = 12.3, 5.5 Hz, 1H), 3.55 (q, *J* = 9.9 Hz, 1H), 2.43 (dt, *J* = 13.2, 7.2 Hz, 1H), 2.27 – 2.20 (m, 1H), 1.62 (s, 9H). Data is in accordance with the direct irradiation pathway result.

2.8 Synthesis of core-shell-shell UCNP (NaYF_4_ @NaYF_4_:75%Yb^3+^,0.5%Er^3+^@NaYF_4_)

UCNP was synthesized in three steps. The first step was the synthesis of β-NaYF_4_ core following the reported co-precipitation approach.^[7]^ In a typical procedure, YCl_3_•6H_2_O (1 mmol, 303.36 mg) was added to a 100 mL three-neck round-bottom flask with 6 mL OA and 15 mL ODE. The mixture solution was heated up to 140 °C in vacuum and stirring for 30 min to form lanthanide oleate complexes. Followed by cooling down to room temperature and addition of 13 mL NaOH/NH_4_F (2.5 mmol and 4 mmol respectively) methanol solution. The mixture was then stirred for another 30 min, before heating up to 75 °C to remove the methanol. The mixture was afterwards slowly heated to 150 °C in vacuum to remove residual water followed by maintaining at 300 °C under N_2_ flow for 90 min before cooling down to room temperature. After washing once with acetone and twice with ethanol, the β-NaYF_4_ bare core nanoparticles were redispersed in 4 mL cyclohexane.

The second step was the synthesis of NaYF_4_ @NaYF_4_:75%Yb^3+^,0.5%Er^3+^ core-active shell structure following successive one-pot layer-by-layer (SLBL) protocol.^[8]^ Here, 1 mL of the above prepared bare core in cyclohexane (NaYF_4_, 0.25 mmol) was added into a 100 mL three-neck round-bottom flask followed by 3 mL OA and 7.5 mL ODE addition. The solution was then heated up to 100 °C in vacuum for 30 min to remove cyclohexane and residual air. After that, the solution was heated up to 300 °C under N_2_ atmosphere and stirred for 5 min. The as-obtained NaYF_4_: 75% Yb^3+^, 0.5% Tm^3+^ shell precursors (10 mL) were injected into the solution in four 15-min intervals. After injection of all shell precursors, the mixture was allowed to ripen for 45 min before cooling down. The core-shell UCNPs (NaYF_4_ @ NaYF_4_:75%Yb^3+^,0.5%Er^3+^) were collected by centrifugation with acetone once and ethanol twice and redispersed in 5 mL cyclohexane.

The third step was the synthesis of NaYF_4_ @ NaYF_4_:75%Yb^3+^,0.5%Er^3+^@NaYF_4_ core-active shell-inert shell structure following the same approach as the one for core-shell structure. A sample of 2 mL core-shell UCNP in cyclohexane (NaYF_4_ @ NaYF_4_:75%Yb^3+^,0.5%Er^3+^, 0.5mmol) was added into a 100 mL three-neck round-bottom flask followed by 3 mL OA and 7.5 mL ODE addition. The solution was then heated up to 100 °C in vacuum for 30 min to remove cyclohexane and residual air. Subsequently, the solution was heated up further to 300 °C under N_2_ atmosphere and stirred for 5 min. The as-obtained NaYF_4_ shell precursors (5 mL) were injected into the solution in two 15-min intervals. After injection of all shell precursors the mixture was allowed to ripen 45 min. The mixture was then cooled down to room temperature and the core-shell-shell UCNP (NaYF_4_ @ NaYF_4_:75%Yb^3+^,0.5%Er^3+^@NaYF_4_) was collected by centrifugation with acetone once and ethanol twice and redispersed in 4 mL cyclohexane.^[6]^

2.9 Synthesis of BODIPY

Acetyl chloride (108 mg, 1.38 mmol) was added to a solution of 2,4-dimethylpyrrole (238 mg, 2.50 mmol) in chloroform (2.5 mL) and the resulting mixture was heated to 50 °C for 1 h. Then, the reaction mixture was cooled in an ice-water bath and Et_3_N (522 µL, 3.75 mmol) was added dropwise, followed by BF_3_OEt_2_ (620 µL, 5.00 mmol). The reaction mixture was stirred at room temperature for 1 h, and then sat. NaHCO_3_ (10 mL) was added, along with DCM (10 mL). The layers were separated, and the aqueous layer was extracted once more with DCM (10 mL). The combined organic layers were dried over MgSO_4_, filtered, and concentrated. The residue was purified by column chromatography over silica gel eluted with toluene/pentane to yield **Methyl- BODIPY** as a dark brown powder (170 mg). ^1^H NMR (400 MHz, *CDCl_3_*) δ = 6.05 (s, 2H), 2.57 (s, 3H), 2.52 (s, 6H), 2.41 (s, 6H). Data is in accordance with the literature.^[5]^

NIS (337 mg, 1.50 mmol) was added to a solution of **Methyl-BODIPY** (157 mg, 0.6 mmol) in dichloromethane (4.8 mL) and hexafluoroisopropanol (HFIP, 1.2 mL) and the solution was cooled in an ice-water bath and stirred for 20 min. Sat. aq. Na_2_S_2_O_3_ (10 mL) was added, along with Et_2_O (20 mL). The layers were separated, and the organic layer was washed once more with sat. NaHCO_3_ (aqueous, 10 mL). The organic layer was separated, dried over MgSO_4_, filtered and concentrated to yield **BODIPY** as a deep red powder (256 mg, 0.50 mmol, 83% yield). ^1^H NMR (400 MHz, *CDCl_3_*) δ = 2.64 (s, 3H), 2.61 (s, 6H), 2.47 (s, 6H). Data is in accordance with the literature.^[5]^

**Scheme S4.** Synthesis of **BODIPY**.

3. NMR spectra

**Figure S1.** ^1^H NMR of **PQ-3TP** in DMSO-*d_6_*

**Figure S2.** ^13^C NMR of **PQ-3TP** in DMSO-*d_6_*

**Figure S3.** ^1^H NMR of **PQ-3CF_3_** in DMSO-*d_6_*

**Figure S4.** ^19^F NMR of **PQ-3CF_3_** in DMSO-*d_6_*

**Figure S5.** ^13^C NMR of **PQ-3CF_3_** in DMSO-*d_6_*

**Figure S6.** ^1^H NMR of **PQ-3OCH_3_** in DMSO-*d_6_*

**Figure S7.** ^13^C NMR of **PQ-3OCH_3_** in DMSO-*d_6_*

**Figure S8.** ^1^H NMR of **PQ-PY** in DMSO-*d_6_*

**Figure S9.** ^1^H NMR of **PQ-PY** in DMSO-*d_6_* (TTA-UC system)

**Figure S10.** ^13^C NMR of **PQ-PY** in DMSO-*d_6_*

**Figure S11.** ^1^H NMR of **PQ-3TP-PY** in DMSO-*d_6_*

**Figure S12.** ^1^H NMR of **PQ-3TP-PY** in DMSO-*d_6_* (TTA-UC system)

**Figure S13.** ^13^C NMR of **PQ-3TP-PY** in DMSO-*d_6_*

**Figure S14.** ^1^H NMR of **PQ-3CF_3_-PY** in DMSO-*d_6_*

**Figure S15.** ^19^F NMR of **PQ-3CF_3_-PY** in DMSO-*d_6_*

**Figure S16.** ^1^H NMR of **PQ-3CF_3_-PY** in *CDCl_3_* (TTA-UC system)

**Figure S17.** ^13^C NMR of **PQ-3CF_3_-PY** in DMSO-*d_6_*

**Figure S18.** ^1^H NMR of **PQ-3OCH_3_-PY** in DMSO-*d_6_*

**Figure S19.** ^1^H NMR of **PQ-3OCH_3_-PY** in *CDCl_3_* (TTA-UC system)

**Figure S20.** ^13^C NMR of **PQ-3OCH_3_-PY** in DMSO-*d_6_*

**Figure S21.** ^1^H NMR of **Methyl-BODIPY** in *CDCl_3_*.

**Figure S22.** ^1^H NMR of **BODIPY** in *CDCl_3_*.

4. Photophysical and Photochemical Studies by UV-Visible Spectroscopy, Emission Spectroscopy, nsTA Spectroscopy

**Figure S23.** Log-log plot of the triplet-triplet annihilation upconversion (TTA-UC) intensity versus the excitation power density. The green and orange lines are quadratic and linear fits, respectively, and the crossover point is defined as the TTA-UC threshold (I_th_).

**Figure S24.** The kinetics of the **PdOEP** at 670 nm before (orange) and after (purple) adding **DPA** under 532 nm excitation.

**Figure S25.** TA kinetics of **PdOEP** at the XB (545 nm) before a) and after b) adding **DPA**.

**Figure S26.** Phosphorescence kinetics of **PdOEP** at the E_m_ (680 nm) before a) and after b) adding **DPA**.

**Figure S27.** TA spectra of a) **PdOEP** and b) **PdOEP** and **DPA** complexes. Excited by 532 nm pulses. XB band (545 nm) and the formation of excited triplet of **DPA** features (~450 nm) were observed.

**Table S3.** Kinetics fitting parameters for UC system.

| Wavelength  (nm) | **PdOEP**  lifetime (μs) | **PdOEP**&**DPA**  lifetime (μs) |
| --- | --- | --- |
| 435 (UCPL) |  | 3.1 (TET)  60.3 (decay) |
| 545 (XB) | 39.4 | 2.3 |
| 670 (T_1_) | 40.1 | 2.3 |
| 680 (E_m_) | 55.1 | 2.4 |

**Figure S28.** Mixed absorption spectra of photoclick (**PQ**, **PY**) and TTA-UC samples (**PdOEP**, **DPA**).

**Figure S29.** a) Physical photograph of TTA-UC driven photoclick reaction (directly mixed system) under the irradiation of a 550 nm LED light source. A magnetic stir bar was added to the mixed sample, along with a long-pass filter to filter the wavelengths below 550 nm. b) Physically separated setup: an inner tube containing the upconversion mixture (**PdOEP**/**DPA**) was placed inside a quartz cuvette containing the reaction substrates (**PQ**s/**PY**). Green light irradiated from the bottom, and the upconverted blue emission from the inner tube propagates into the outer compartment to drive the reaction.

**Figure S30.** The UCPL lifetime of **DPA** as a function of **PQ**s concentration (0-200 μM).

**Figure S31.** a) Absorption spectra of **PQ**s with varied concentrations in the TTA-UC solution; b) UCPL quenching with addition of different concentration of **PQ**s (0-200 μM); c) Stern-Volmer plot constructed from the steady-state UCPL (I_0_/I - 1 vs. [**PQ**s]), exhibiting a pronounced upward curvature that is characteristic of radiative energy transfer (reabsorption/inner-filter effect) and inconsistent with a FRET-dominated mechanism.

**Figure S32.** UCQY and pseudo-first-order kinetics (*k_obs_*) various different sensitizer/annihilator concentration ratio.

**Figure S33.** Time-resolved UV-vis absorption spectra of **PQ-ERA** photoclick reactions under 365 nm irradiation. a) Photocycloaddition between **PQ** and **PY**; b) **PQ-3TP** and **PY**; c) **PQ-3OCH_3_** and **PY**; and d) **PQ-3CF_3_** and **PY**.

**Figure S34.** Color change of the **PQ_PY** photoclick samples before and after irradiation.

**Figure S35.** Absorption spectra of **PQ** and **PY** mixed with a) **PdOEP** and b) **DPA** samples under 550 nm LED irradiation over time.

**Figure S36.** Kinetic traces for photocycloaddition between **PQ_PY** and TTA-UC sample containing dissolved oxygen.

**Figure S37.** a) Time-resolved UV-vis absorption spectra of a mixture of **PQ‑3TP**, **DHF**, **PdOEP**, and **DPA** under 550 nm LED irradiation, showing progressive product formation at ~310 nm. b) Kinetic trace at 430 nm extracted from a).

**Figure S38.** Time-resolved UV-vis absorption spectra of **PQ-3TP** (50 μM) and **PY** (500 μM) mixed with a) **PdOEP** (30 μM) and b) **DPA** (250 μM) samples under 550 nm LED irradiation over time. No detectable product formation is observed in either case.

**Figure S39.** a) Time-resolved UV-vis absorption spectra and b) kinetic traces at 430 nm of **PQ-3TP** (50 μM) and **PY** (500 μM) in the presence of UCNPs (1.5 mg/mL) under 550 nm LED irradiation. No detectable product formation is observed.

**Scheme S5.** Jablonski diagram illustrating the TTA‑UC mechanism between the **BODIPY** sensitizer and perylene annihilator.

**Figure S40.** a) Normalized absorption and photoluminescence (PL) spectra of **BODIPY**. b) Normalized absorption and PL spectra of perylene. c) Corrected UCPL spectrum of the **BODIPY**/perylene mixture (4 μM/320 μM) under 532 nm excitation. The spectrum is corrected for the inner filter effect arising from the high perylene concentration. d) Normalized absorption spectrum of **PQ‑3TP** and the corrected UCPL emission spectrum, showing spectral overlap.

**Figure S41.** a) Time-resolved UV-vis absorption spectra of a mixture of **PQ‑3TP**, **PY**, **BODIPY**, and perylene under 550 nm LED irradiation, showing progressive product formation at ~310 nm. b) Kinetic trace monitored at 332 nm extracted from a).

**Figure S42.** Time-resolved UV-vis absorption spectra of the photoclick reaction occurred in physically separated TTA-UC system shown in Figure S29b. a) Photocycloaddition between **PQ** and **PY**; b) Photocycloaddition between **PQ-3TP** and **PY**; c) Photocycloaddition between **PQ-3OCH_3_** and **PY**; d) Photocycloaddition between **PQ-3CF_3_** and **PY**. The spectral evolution shows progressive product formation, confirming that the reaction is driven solely by upconverted blue photons.

**Figure S43. PEG**-encapsulated aqueous TTA-UC photoclick system. a) Chemical structure of **PEG**; b) Schematic illustration of the encapsulated **PdOEP**/**DPA** system and **PQ**s/**PY** reactants dispersed in water; c) Time-resolved UV-vis absorption spectra of the aqueous reaction mixture under 550 nm LED irradiation, showing progressive product formation at ~310 nm, the inset enlarges the 300-380 nm region, where equimolar absorption point indicate a clean conversion from reactants to product without significant side reactions; d) Kinetic trace at 310 nm extracted from c). The steady absorbance increase confirms efficient aqueous-phase green-light-driven photoclick reactivity.

**Figure S44.** Time-resolved UV-vis absorption spectra of the aqueous reaction mixture under 550 nm LED irradiation in a) pH 6 buffer, b) pH 9 buffer, and c) hydrogel. Inset: photograph of the **PQ‑ERA** solution in hydrogel. d) Kinetic trace at 315 nm extracted from a). e) Kinetic trace at 430 nm extracted from b). f) Kinetic trace at 430 nm extracted from c).

Reference

[1]	Q. Luan, L. Hou, H. Li, T. Wang, T. Xu, Z. Ding, J. Jiang, T. Liu, Measurement methods for triplet-triplet annihilation photon upconversion*,* *Opt. Continuum* **2024**, *3*, 2078-2087.

[2]	M. Fischer, J. Georges, Fluorescence quantum yield of rhodamine 6G in ethanol as a function of concentration using thermal lens spectrometry*,* *Chem. Phys. Lett.* **1996**, *260*, 115-118.

[3]	G. A. Crosby, J. N. Demas, Measurement of photoluminescence quantum yields. Review*,* *J. Phys. Chem.* **1971**, *75*, 991-1024.

[4]	N. Yanai, K. Suzuki, T. Ogawa, Y. Sasaki, N. Harada, N. Kimizuka, Absolute Method to Certify Quantum Yields of Photon Upconversion via Triplet–Triplet Annihilation*,* *J. Phys. Chem. A* **2019**, *123*, 10197-10203.

[5]	Y. Fu, G. Alachouzos, N. A. Simeth, M. Di Donato, M. F. Hilbers, W. J. Buma, W. Szymanski, B. L. Feringa, Triplet-Triplet Energy Transfer: A Simple Strategy for an Efficient Visible Light-Induced Photoclick Reaction*,* *Angew. Chem. Int. Ed.* **2024**, *63*, e202319321.

[6]	Y. Fu, K. Wu, G. Alachouzos, N. A. Simeth, T. Freese, M. Falkowski, W. Szymanski, H. Zhang, B. L. Feringa, Efficient, Near‐Infrared Light‐Induced Photoclick Reaction Enabled by Upconversion Nanoparticles*,* *Adv. Funct. Mater.* **2023**, *33*, 2306531.

[7]	H.-S. Qian, Y. Zhang, Synthesis of Hexagonal-Phase Core−Shell NaYF4 Nanocrystals with Tunable Upconversion Fluorescence*,* *Langmuir* **2008**, *24*, 12123-12125.

[8]	X. Li, D. Shen, J. Yang, C. Yao, R. Che, F. Zhang, D. Zhao, Successive Layer-by-Layer Strategy for Multi-Shell Epitaxial Growth: Shell Thickness and Doping Position Dependence in Upconverting Optical Properties*,* *Chem. Mater.* **2012**, *25*, 106-112.
